# Antigen Presenting Cells and Stromal Cells Trigger Human Natural Killer Lymphocytes to Autoreactivity: Evidence for the Involvement of Natural Cytotoxicity Receptors (NCR) and NKG2D

**DOI:** 10.1080/17402520600578194

**Published:** 2006

**Authors:** Alessandro Poggi, Maria Raffaella Zocchi

**Affiliations:** 1 National Institute for Cancer Research Genoa 16132 Italy; 2 San Raffaele Scientific Institute Milan 20132 Italy

## Abstract

Human natural killer (NK) lymphocytes should not damage autologous cells due to the engagement of inhibitory receptor superfamily (IRS) members by HLA-I. Nevertheless, NK cells kill self cells expressing low levels or lacking HLA-I, as it may occur during viral infections (missing-self hypothesis). Herein, we show that human NK cells can be activated upon binding with self antigen presenting cells or stromal cells despite the expression of HLA-I. Indeed, NK cells can kill and produce pro-inflammatory and regulating cytokines as IFN-γ, TNF-α and IL10 during interaction with autologous dendritic cells or bone marrow stromal cells or skin fibroblasts. The killing of antigen presenting and stromal cells is dependent on LFA1/ICAM1 interaction. Further, the natural cytotoxicity receptors (NCR) NKp30 and NKp46 are responsible for the delivery of lethal hit to DC, whereas NKG2D activating receptor, the ligand of the MHC-related molecule MIC-A and the UL16 binding protein, is involved in stromal cell killing. These findings indicate that different activating receptors are involved in cell to self cell interaction. Finally, NK cells can revert the veto effect of stromal cells on mixed lymphocyte reaction further supporting the idea that NK cells may alter the interaction between T lymphocytes and microenvironment leading to autoreactivity.


*Clinical & Developmental Immunology*, June–December 2006; 13(2–4): 325–336

Taylor & Francis

Taylor & Francis Group

### Antigen presenting cells and stromal cells trigger human natural killer lymphocytes to autoreactivity: Evidence for the involvement of natural cytotoxicity receptors (NCR) and NKG2D

ALESSANDRO POGGI[1] & MARIA RAFFAELLA ZOCCHI[2]

[1]*National Institute for Cancer Research, 16132 Genoa, Italy, and* [2]*San Raffaele Scientific Institute, 20132 Milan, Italy*

**Abstract**
Human natural killer (NK) lymphocytes should not damage autologous cells due to the engagement of inhibitory receptor superfamily (IRS) members by HLA-I. Nevertheless, NK cells kill self cells expressing low levels or lacking HLA-I, as it may occur during viral infections (missing-self hypothesis). Herein, we show that human NK cells can be activated upon binding with self antigen presenting cells or stromal cells despite the expression of HLA-I. Indeed, NK cells can kill and produce pro-inflammatory and regulating cytokines as IFN-γ, TNF-α and IL10 during interaction with autologous dendritic cells or bone marrow stromal cells or skin fibroblasts. The killing of antigen presenting and stromal cells is dependent on LFA1/ICAM1 interaction. Further, the natural cytotoxicity receptors (NCR) NKp30 and NKp46 are responsible for the delivery of lethal hit to DC, whereas NKG2D activating receptor, the ligand of the MHC-related molecule MIC-A and the UL16 binding protein, is involved in stromal cell killing. These findings indicate that different activating receptors are involved in cell to self cell interaction. Finally, NK cells can revert the veto effect of stromal cells on mixed lymphocyte reaction further supporting the idea that NK cells may alter the interaction between T lymphocytes and microenvironment leading to autoreactivity.

**Keywords:** *Natural killer lymphocyte, inhibitory receptor superfamily, HLA-I, TNF-α*

### Introduction

The adaptive immune response represents an important component of host defence against infections and thus critical for health (Janaway 1992). However, adaptive immune responses sometimes are elicited against self-antigens and this promotes severe diseases (Janaway 1992, Goodnow 1996, lanzavecchia and Sallusto 2001, von Herrath and Harrison 2003). Normally, self-tolerance mechanisms prevent autoimmune disease: It is thought that these mechanisms take place both within the thymus and in the periphery. The main target of these mechanisms is the immunocompetent cell represented by B and T lymphocytes, which can recognize the antigen via their antigen receptor. Several clues are used by the immune system to distinguish self ligands from non-self ligands. Indeed, an ineffective immune response could occur when the ligand is encountered by an immature lymphocyte leading to death or to inactivation. Alternatively, the presence of high and stable concentration of ligand (as it happens for self-antigens) can result in tolerance; further, the absence of co-stimulatory signals may impair lymphocyte response to self-ligands. In these tolerizing processes, as well as during adaptive immune response against non-self antigens the innate arm of the immune system play a key role (Janaway 1992). Dendritic cells (DC) that are considered a bridge between innate and acquired immunity, are specialized antigen presenting cells (APC) of myeloid origin responsible for the adequate antigen presentation and for T lymphocyte activation (Lanzavecchia and Sallusto 2001); the lack of co-stimulatory molecules on DC determine an abortive immune response leading to tolerance. It is conceivable that perturbation of these processes may lead to undesired events as the triggering of autoimmune diseases.

Among the various effectors cells of the innate arm of immune system natural killer (NK) lymphocytes may influence the outcome of adaptive immune

Correspondence: A. Poggi, Laboratory of Experimental Oncology D, National Institute for Cancer Research, c/o CBA Torre A1, Largo R. Benzi 10, 16132 Genoa, Italy. Tel: 39 0105737211. Fax: 39 010354282. E-mail: alessandro.poggi@ostge.it

ISSN 1740-2522 print/ISSN 1740-2530 online © 2006 Taylor & Francis
DOI: 10.1080/17402520600578194

response by interacting with either APC or epithelial as well as stromal cells. In this review, we will analyse this interaction and discuss the consequences with respect to initiate autoimmune reaction.

### Results and discussion

### *Human NK cells recognize self-HLA class I through the engagement of inhibitory receptors superfamily (IRS)*

Human NK cells play a role in eliminating virus-infected cells as well as in controlling tumor cell growth (Trinchieri 1989). This lymphocyte population originate from bone marrow where it resides and recirculate from the peripheral blood. NK cells are the most cytolytic effector lymphocytes, which can kill target cells without prior sensibilization. By contrast with cytolytic T lymphocytes, NK cells apparently recognize targets lacking HLA class I and peptide. Paradoxically, NK cell-mediated activities are down regulated by interacting with HLA class I (Figure 1A). Indeed, NK lymphocytes are characterized by the surface expression of receptors for self-HLA class I molecules including some members of the inhibitory receptor superfamily (IRS), whose engagement leads to the inhibition of cytolysis and cytokine production. Thus, according to the missing self hypothesis, NK cells can kill targets that do not express (or express low levels) of HLA class I, such as tumor and virus-infected cells (Figure 1C) (Farag et al. 1947, Ljunggren and Karre 1990, Moretta et al. 1996, Lanier 1998, Long 1999).

According to this hypothesis, NK cells do not exert any activity against self as usually host tissues express HLA class I. However, this is in contrast with the finding that, although some tissues do not bear HLA class I, they are not damaged by NK cells. In addition, the discovery of isoforms of IRS that, upon recognition of HLA class I, deliver an activating signal in NK cells that leads to the triggering of cytolytic activity and cytokine release indicate that NK cells can be actually activated by self-HLA class I (Figure 1) (Farag et al. 1947, Moretta et al. 1996, Lanier 1998, Long 1999). To conciliate these conflicting findings it has been stated that the negative signal delivered via inhibiting IRS always overcomes the activating ones when these two opposite signals take place in close proximity. Alternatively, it has been shown that the affinity of inhibiting forms for a given HLA class I is higher than that of activating IRS (Farag et al. 1947, Moretta et al. 1996, Lanier 1998, Long 1999). However, as both activating and inhibiting IRS are clonally distributed and each IRS can recognize one or a limited number of HLA class I alleles it is possible

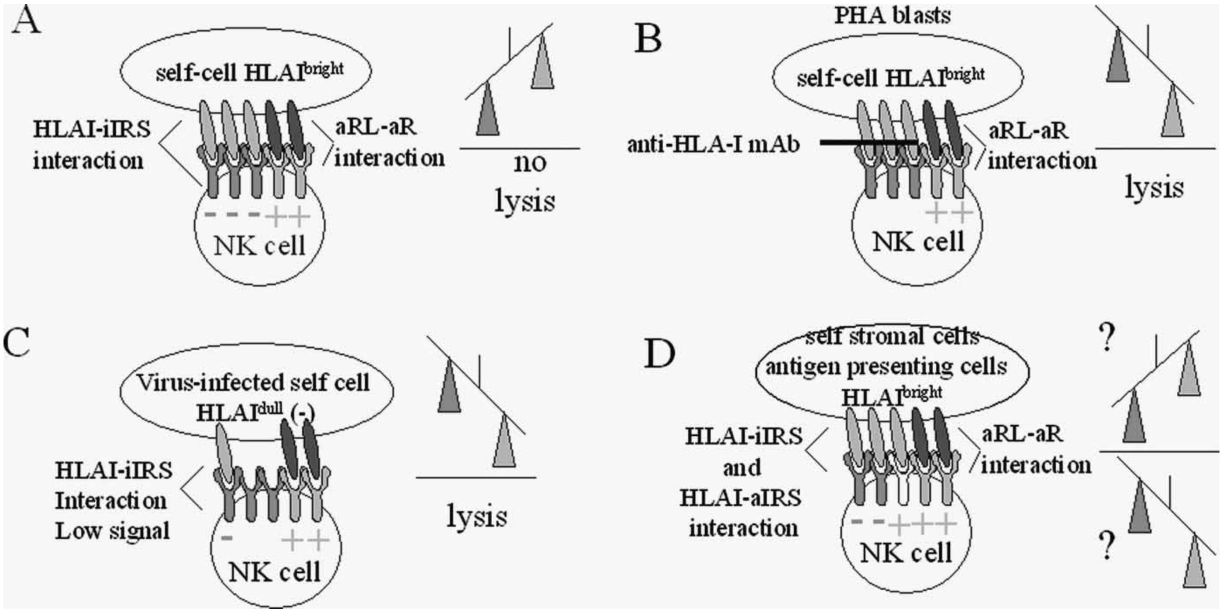

Figure 1. Missing self-hypothesis to explain the lack of NK cell aggression to autologous cells. NK cells bear at the cell surface members of the IRS of the inhibiting type (iIRS) recognizing self-HLA-I: iIRS engagement delivers an inhibiting signal that overcomes the activating one initiated through activating receptors (aR)-activating receptor ligand (aRL) interaction (A); this results in absence of lysis. This inhibiting signal is blocked by the addition of anti-HLAI mAb to the cytolytic assay (B) that impair HLA-I-iIRS interaction; this results in the reconstitution of lytic effect. Panel (C). The lysis of self-cell expressing low levels (or lacking) HLA-I can occur because the inhibiting signal does not overcome the activating one. Panel (D). This scenario is complicated by the presence, in any healthy donor, of NK cells with activating isoforms of IRS (aIRS): Their engagement with self-HLA-I leads to activation of NK cell-mediated cytolysis and cytokine release. Thus, the outcome of NK cell-self cell interaction would be the result of the balance between activating and inhibiting signals depending on the expression and physical localization of aIRS, iIRS, aR and aRL. In each panel, the balance between activation (green) and inactivation of lysis (red) is shown. In red are depicted the inhibitory receptors, in green the activating ones. Yellow receptors are the IRS with activating function.

that some NK cells bear an activating IRS and an inhibiting one for different HLA class I alleles; this implies that, to overcome the activating signals, inhibiting IRS are engaged together activating IRS by the corresponding HLA class I allele and this is far from to be demonstrated. In addition, it should be noted that the large majority of experimental data supporting the idea that HLA class I protect self cells from NK cell-mediated injury, derives from the finding that monoclonal antibodies (mAb) to HLA class I and/or to inhibiting IRS strongly enhance the lytic activity of NK cells when added to a cytolytic assay using phytohemoagglutinin (PHA) activated lymphocytes as targets (Figure 1B) (Farag et al. 1947, Ljunggren and Karre 1990, Moretta et al. 1996, Lanier 1998, Long 1999). Few data are reported using target cells from tissues other than peripheral blood. Further, APC (Carbone et al. 1999, Wilson et al. 1999, Parajuli et al. 1999, Spaggiari et al. 2001, Ferlazzo et al. 2002, Pende et al. 2005) cells have been reported to be optimal targets for NK cell-mediated killing, indicating on one hand that the protecting role of HLA class I is relevant in particular for lymphocytes and on the other hand that additional receptors may play a role in distinguishing between self and non-self (Figure 1D).

### *Interaction between NK cells and APC: Role of natural cytotoxicity receptors (NCR)*

Autologous IL2 activated NK cell populations are able to lyse freshly isolated APC including monocytes or monocyte-derived DC cultured for 7 days with appropriate combination of cytokines as GM-CSF and IL4 and/or TNF-α which are known to induce monocyte maturation toward DC. As shown in Figure 2, the NK cell-mediated lysis of self-APC is stronger against DC cultured with GM-CSF, IL4 and TNF-α compared to APC obtained with GM-CSF alone or in combination with IL4 or *ex vivo* isolated monocytes (Spaggiari et al. 2001). On the other hand, resting NK cells are not able to kill self-APC, although they efficiently lysed K562 tumor cells line (Figure 2A). That NK cells can damage APC was further confirmed by the analysis of a series of NK cell clones. Indeed, two groups of NK cell clones could be identified on the basis of the degree of APC lysis: the first group strongly killed self-APC while the

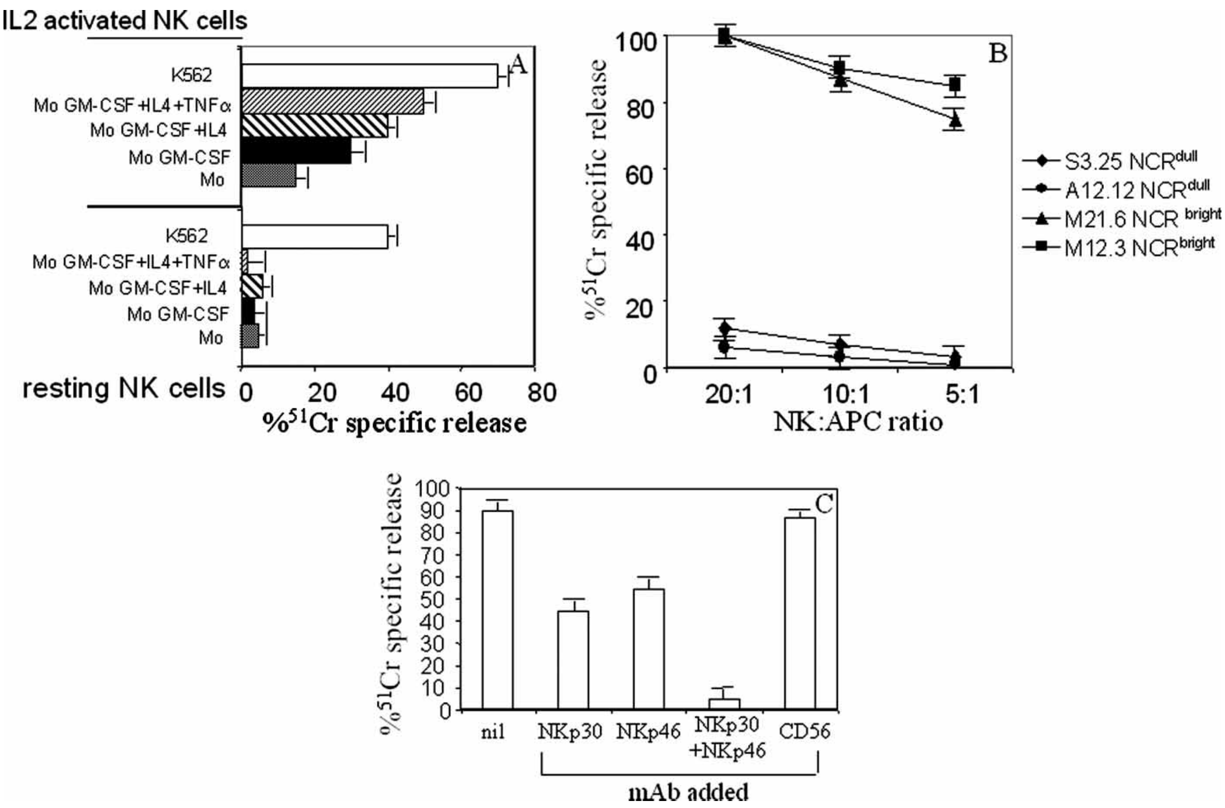

Figure 2. NK cell-mediated lysis of APC is initiated via the engagement of NKp30 and NKp46. Panel (A). IL2-activated NK cells lyse APC such as monocytes (Mo) or as monocytes cultured with differentiating cytokines, whereas *ex vivo* isolated NK cells do not. Panel (B). This effect is strictly related to the expression of NKp30 and NKp46 (NCR) activating receptors. The two representative NK cell clones M21.6 and M12.3 bearing a bright expression of NKp30 and NKp46 killed self-APC, whereas the S3.25 and A12.12 $NCR^{dull}$ NK cell clones did not. Panel (C). The addition of anti-NKp30 and/or NKp46 mAb strongly inhibited the lysis of APC exerted by NK cells. Results are expressed as % $^{51}Cr$ specific release in a 4 h cytolytic assay at effector (E, NK cell) target (T, self APC) ratio of 10:1 (A) 20:1, 10:1, 5:1 (B) and 10:1 (C), respectively. Results obtained with anti-CD56 mAb are shown for comparison.

second group did not. Importantly, NK cell clones which efficiently lysed self APC expressed high levels of the recently identified NCR (Moretta et al. 2001) NKp30, NKp44 and NKp46 activating receptors at the cell surface; by contrast, a low expression (10-fold lower) of these receptors was found on NK cell clones which do not lyse APC (Figure 2B). This finding suggests that the expression of NCR directly correlates with APC killing. To determine whether these receptors are actually involved in NK cell mediated killing of APC we performed some experiments in the presence of anti-NCR monoclonal antibodies. Indeed, it has been shown that the covering of these receptors with specific anti-NCR mAb leads to the inhibition of cytolysis of several tumor targets as a consequence of the lack of interaction of NCR (Moretta et al. 2001), expressed on NK cells, with their putative ligand(s) on target cells. As shown in Figure 2C, the addition of anti-NKp30 or anti-NKp46 mAb to the cytolytic assay reduced by approximately 50% the lysis of autologous APC. Further, the addition of anti-NKp30 and anti-NKp46 mAb in combination led to a complete inhibition of APC lysis. This indicates that these two NCRs are the surface molecular structures involved in the delivering of the lethal hit to APC (Spaggiari et al. 2001). To determine the biochemical mechanism by which NK cell clones lyse self-APC via the engagement of NKp30 and NKp46, cytolytic assay were performed in the presence of the phosphatidyl-inositol-3 kinase inhibitors wortmannin (irreversible) or LY294002 (reversible). Indeed, APC lysis was strongly inhibited (up to 80%) by blocking PI-3K with micromolar concentrations of LY294002 or nanomolar concentrations of wortmannin (Figure 3A). The engagement of NKp30 or NKp46 with specific mAbs leads to the activation of Akt/PKB, which is a specific PI-3K substrate (Figure 3B) and the same PI-3K inhibitors impaired the killing in a re-directed killing assay of the Fc$\gamma$R$^+$ target cell P815 initiated via NKp30 or NKp46 (not shown). Altogether, these findings strongly suggest that the molecular mechanism underlying the lysis of autologous APC by NK cells is linked to the PI-3K/Akt signal transduction pathway (Spaggiari et al. 2001).

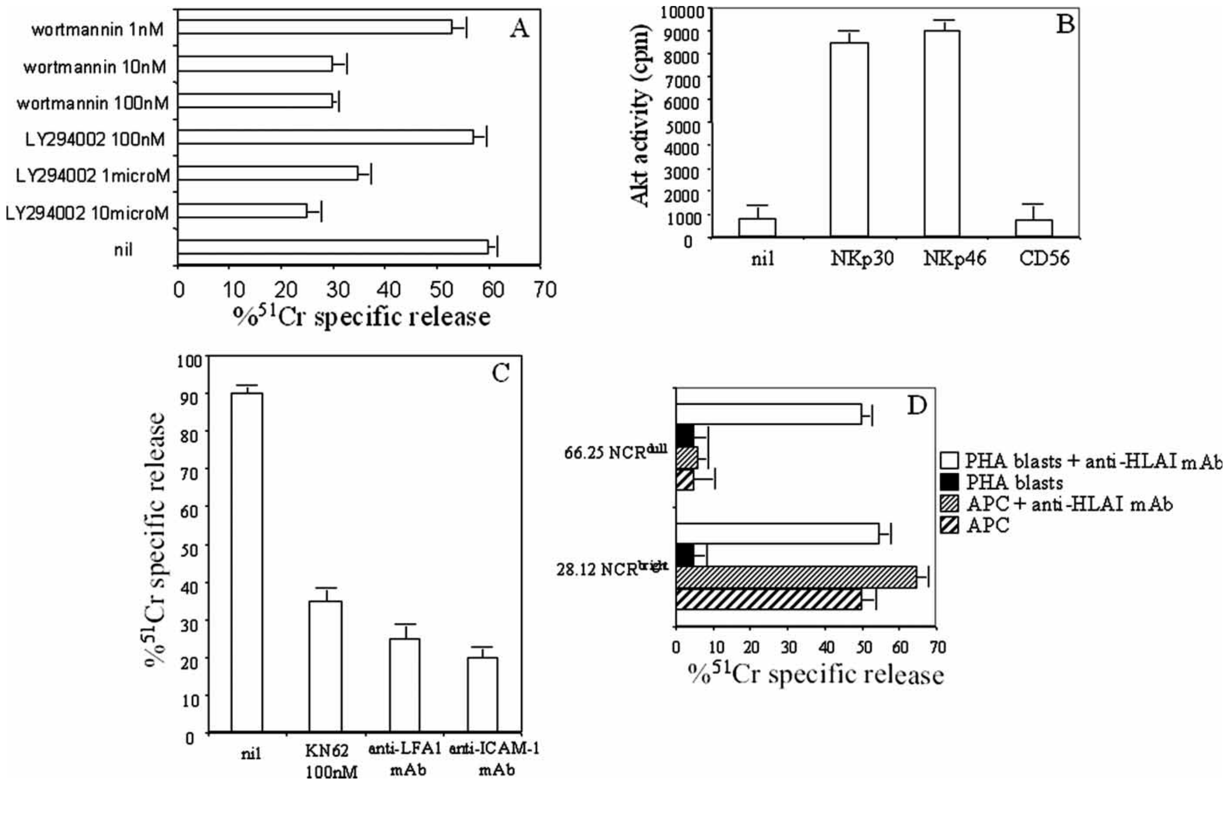

Figure 3. Involvement of PI-3K and CAMKII in NK-APC interaction. Lack of the protective effect by HLAI. Panel (A). Lysis of APC was inhibited in a dose dependent manner by the addition of the PI-3K inhibitors wortmannin or LY294002. Panel (B). Activity of Akt/PKB, a downstream substrate of PI-3K, is enhanced by the engagement of NKp30 or NKp46. Panel (C). Lysis of APC is inhibited by the CAMKII inhibitor KN62 and by the addition of anti-LFA1 or anti-ICAM1 mAb. Panel (D). The covering of HLA-I does not enhance the lysis of APC whereas self-PHA blasts, as expected, are lysed only in the presence of anti-HLA-I mAb. Results in panel (A), (C) and (D) are expressed as % $^{51}$Cr specific release at the E/T ratio of 10:1. Results in panel (B) are expressed as Akt activity (cpm) using the specific substrate and $\gamma$-$^{32}$P ATP after immunoprecipitation with anti-Akt specific mAb. Akt activity was analyzed at different time points (1, 3, 5 min) and data presented referred to min 3 of stimulation with medium (nil) or mAb to the indicated molecules followed by goat anti-mouse cross-linking.

*Role of LFA1-ICAM1 interaction in APC lysis by NK lymphocytes*

Lysis of target cells by NK cells requires a number of steps: Target cell binding, calcium entry and release of lytic enzymes (Trinchieri 1989, Leibson 1997, Moretta et al. 2001, Ferlazzo et al. 2002, Pende et al. 2005). Among the surface structures involved in effector-target cell interaction LFA1 play a key role in cell–cell adhesion as well as in delivering an activating signal. Thus, we further investigated which role may have LFA1 in NK cell-mediated APC lysis. It has been demonstrated that the engagement of LFA1 on NK cells can induce calcium influxes through L-type calcium channels and that calcium is needed to deliver the lethal hit (Zocchi et al. 1998). Thus, the interaction between NK and APC evoked a strong calcium influx accompanied by the activation of the calcium-calmodulin kinase II (CAMKII) which can be blocked by the CAMKII inhibitor KN62 and masking LFA1, with the $F(ab')_2$ of the specific mAb (or anti-ICAM1 mAb), conceivably inhibiting cell to cell contact (Figure 3C). Further, the oligomerization of LFA1 elicited by the addition of goat anti-mouse antiserum on NK cells preincubated with anti-LFA1 mAb induced a strong activation of CAMKII (not shown) this close relates to the inhibition of APC lysis exerted by CAMKII inhibitors and anti-LFA1 mAb. Altogether, these findings suggest that during NK cell to APC binding the activation of CAMKII initiated via LFA1 is responsible for the consequent killing of self APC (Poggi et al. 2002).

*Role of HLA-class I in NK cell-mediated lysis of APC*

To determine the role of HLA class I in the lysis of self APC, we analyzed a panel of NK cell clones for their cytolytic activity in the presence of anti-HLA class I specific mAb. As shown in Figure 3D the $NCR^{bright}$ 28.12 NK cell clone lysed APC but not PHA blasts. The covering of the HLA class I molecules slightly increased the lysis of APC but strongly enhanced that of PHA blasts indicating that members of the IRS are actually playing an inhibitory effect when PHA blasts are used as target cells. Further, a similar increase of PHA blasts lysis in the presence of anti-HLA class I mAb was observed using the $NCR^{dull}$ 66.25 NK cell clone which do not lyse APC (Spaggiari et al. 2001). Although not shown, similar results were obtained using anti-IRS mAbs. These findings together with the observation that APC lysis was enhanced by APC treatment with TNF-α, which actually upregulates HLA class I expression, strongly suggest that the inhibiting signal initiated through the IRS-HLA class I engagement do not overcome the NCR-mediated activating signal (Spaggiari et al. 2001). However, we cannot exclude that the TNF-α treatment of APC upregulates, beside HLA class I, also the still undefined ligands of NKp30 and NKp46 thus favouring the activation lytic machinery.

*Phenotypic and functional characterization of bone marrow stromal cells (BMSC)*

Apparently, only IL2 activated NK cells lyse self-APC (Carbone et al. 1999, Wilson et al. 1999, Parajuli et al. 1999, Spaggiari et al. 2001, Ferlazzo et al. 2002, Pende et al. 2005) suggesting that physiologically this killing occurs at the interface between host and the outer environment, for instance at the mucosal site during viral infections when NK cells are activated. However, NK cells reside in the bone marrow and conceivably can interact with bone marrow stromal cells (BMSC) (Trinchieri 1989). BMSC are thought to have a role in the regulation of hemopoietic stem cell proliferation and differentiation as well as in the growth of some hemopoietic malignancies (Rodriguez Mdel et al. 2004, Podesta et al. 2005, Vodyanik et al. 2005). More recently, it has been reported that within the bone marrow stroma are present the mesenchymal stem cells (MSC) (Pittenger et al. 1999, Sekiya et al. 2002). MSC are characterized by the ability of differentiating into mesodermic tissues or even into epithelial cells and neurons (Woodbury et al. 2000). MSC can exert a tolerogenic effect on T lymphocyte activation and proliferation through the release of immunoregulating cytokines as transforming growth factor β (TGFβ) and hepatocyte growth factor (HGF) or indoleamine 2,3 dioxygenase; moreover, T lymphocyte-MSC contact can lead to T-cell tolerance (Di Nicola et al. 2002, Tse et al. 2003, Le Blanc 2003, Potian et al. 2003, Djouad et al. 2003, Meisel et al. 2004, Beyth et al. 2005, Aggarwal and Pittenger 2005). This supports the use of MSC to favour bone marrow engraftment and avoid graft-versus-host disease (GVHD) (Le Blanc et al. 2004). As it has been reported that MSC can cooperate with immunosuppressive drugs to ameliorate GVHD, it is important to determine whether the innate arm of the immune system may interact with BMSC and/or MSC and the consequence of this interaction. Bone marrow cell suspensions were isolated and cultured according to previous reports (Di Nicola et al. 2002, Le Blanc 2003, Potian et al. 2003, Djouad et al. 2003, Meisel et al. 2004, Beyth et al. 2005, Aggarwal and Pittenger 2005) and BMSC were obtained after 15–21 days of culture. At this time surface phenotype of adherent cells was the following: 99%SH3/$CD73^+$ $SH4^+$ SH2/$CD105^+$ 100%$CD44^+$ β1-integrin$(CD29)^+$ ICAM1$(CD54)^+$ 100%HLA-$I^+$ and $CD40^+$, 98%$CD45^-$ $CD31^-$ $CD34^-$ $CD33^-$ $CD3^-$ $CD2^-$ $CD16^-$ $CD14^-$ $ICAM2^-$ $ICAM3^-$ $CD80^-$ $CD86^-$ $CD83^-$ HLA-$DR^-$. Microscopic analysis revealed two types of cells (Figure 4A):

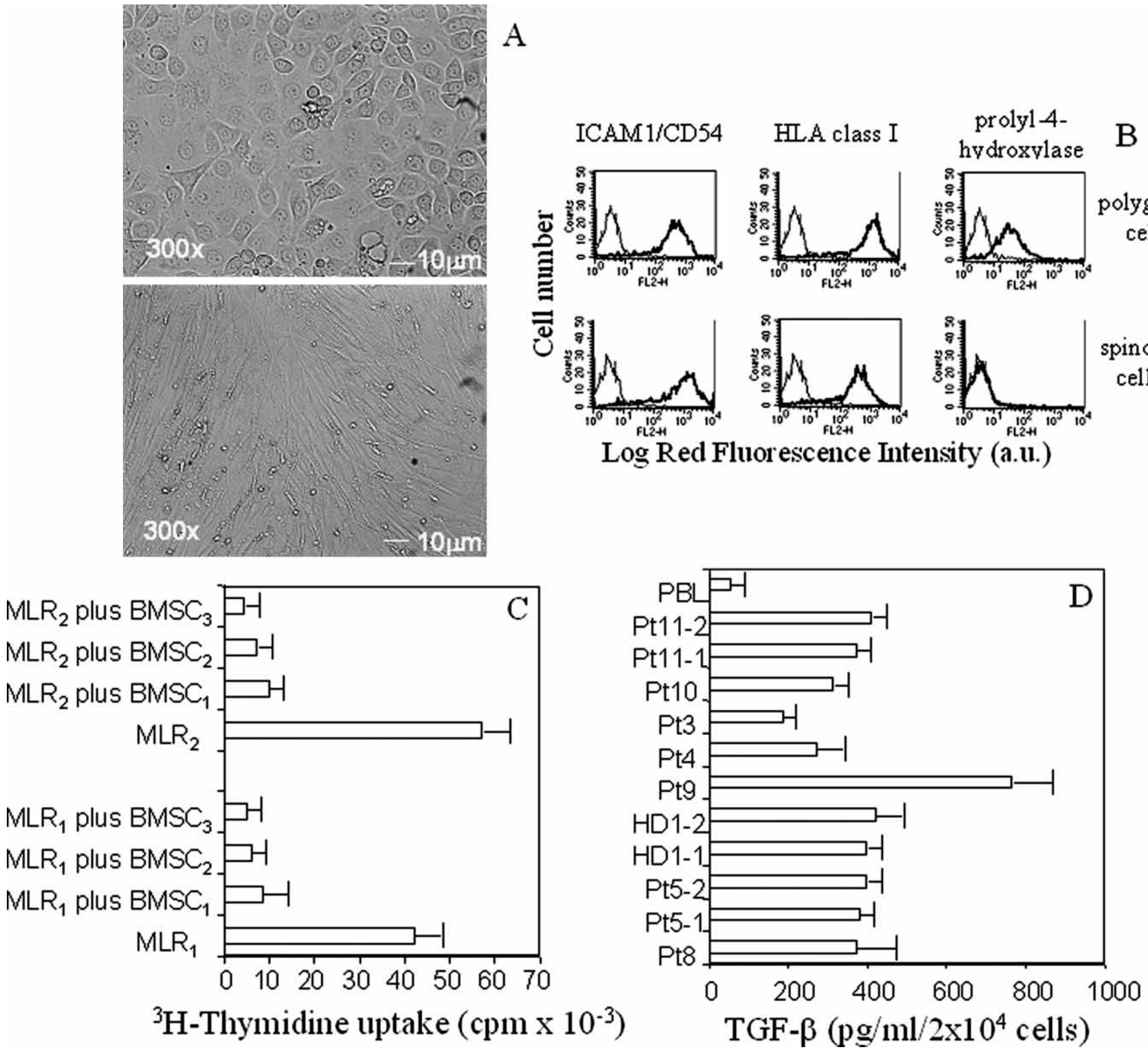

Figure 4. Morphological, phenotypic and functional characterization of BMSC. Panel (A). Culture of BMSC was analyzed by inverted microscope (300 × magnification). Upper quadrant; polygonal shaped BMSC on 14d. Lower quadrant: Spindle shaped BMSC on 14d. Panel (B). Polygonal shaped BMSC (upper histograms) or spindle shaped BMSC (lower histograms) were analyzed for the expression of the indicated receptors by indirect immunofluorescence. Thin grey histogram; staining of cells with an unrelated isotype matched mAb. Bold black histogram; staining with mAb to the indicated antigen. Results are expressed as mean red fluorescence intensity vs number of cells. Panel (C). Effect of BMSC on mixed lymphocyte reaction (MLR). $2 \times 10^4$ irradiated BMSC of three different donors ($BMSC_1$, $BMSC_2$, $BMSC_3$) were added, as a third party, at the onset of the MLR ($MLR_1$ and $MLR_2$, $10^5$ responder cells vs $10^5$ irradiated stimulator cells) and $^3$H-thymidine uptake was evaluated on 7d. Results are expressed as cpm $\times 10^{-3}$. Panel (D). TGFβ production evaluated by ELISA of BMSC: $2 \times 10^4$ BMSC derived from Pt3, Pt4, Pt5 (Pt5-1: spindle shaped; Pt5-2: polygonal shaped), Pt8, Pt9, Pt10, Pt11 (Pt11-1: spindle shaped; Pt11-2: polygonal shaped; HD1 (HD1-1 spindle shaped; HD1-2 polygonal shaped) Results are expressed as pg/ml/2 $\times 10^4$ cells and are the mean ± SD of triplicate samples.

(1) spindle shaped cells, resembling fibroblast morphology which can be considered "bona fide" MSC as they are able to differentiate into adipocytes, condroblasts or osteoblasts under appropriate culture conditions (Pittenger et al. 1999, Woodbury et al. 2000, Sekiya et al. 2002), and (2) polygonal shaped cells similar to epithelial cells positive for prolyl-4-hydroxilase (4PH) (Figure 4B), a marker of fibroblasts unable to maturate. Both spindle and polygonal stromal cells strongly inhibit (range 50–90%, mean 70%, $n = 6$) the proliferation of T lymphocytes in MLR when added at a MSC/T responder ratio of 1:2–1:5 (Figure 4C). Furthermore, the amount of TGFβ spontaneously released in culture by both types of MSC from the same donor was similar (Figure 4D). Thus, both phenotypic and functional features of MSC obtained under our culture conditions were in agreement with what previously reported (Di Nicola et al. 2002, Tse et al. 2003, Le Blanc 2003, Potian et al. 2003, Djouad et al. 2003, Meisel et al. 2004, Le Blanc et al. 2004, Beyth et al. 2005, Aggarwal and Pittenger 2005).

### *Interaction between* ex vivo *isolated NK cells and BMSC: role of LFA1 and NKp30*

To determine the outcome of NK–BMSC interaction, we isolated *ex vivo* NK cells from peripheral blood or bone marrow of healthy donors. Thus we investigated whether NK–BMSC interaction could lead to activation and/or release of cytokines by NK cells (Poggi et al. 2005). We found that NK cells released IFN-γ and TNF-α together with IL10 (Figure 5A) upon binding with BMSC together with the upregulation of the activation antigen CD69 and the *de-novo* expression of CD25 (not shown). Accordingly, FACS and microscopic analysis revealed that NK cells became blasts when co-cultured with BMSC. The binding between NK and BMSC was needed to induce NK cell triggering as when NK were separated from BMSC by a transwell porous membrane, or by the addition of a combination of anti-FLA1 and anti-ICAM1 mAbs, the amount of IFN-γ and/or TNF-α detected in NK–BMSC co-culture supernatant was negligible or highly reduced, respectively (Figure 5B). Further, the anti-NKp30 mAb, but not anti-NKp46 mAb, inhibited (range of inhibition 65–85%; $n = 4$) cytokine production suggesting that this NK cell activating receptor may recognize on BMSC its counterligand (Figure 5B).

### *Interaction between IL2-activated NK cells and BMSC: role of LFA1 and NKG2D*

BMSC may favour cell proliferation and produce soluble factors involved in myeloid precursor maturation and differentiation (Rodriguez Mdel et al. 2004, Podesta et al. 2005, Vodyanik et al. 2005). Thus, we analysed whether IL2-activated NK cells can affect BMSC survival, like it happens with self APC. Again, IL2-activated NK cells efficiently lysed self-BMSC and this killing was evident at very low effector target cell ratios implying that during the interaction of a single NK cell with one BMSC an

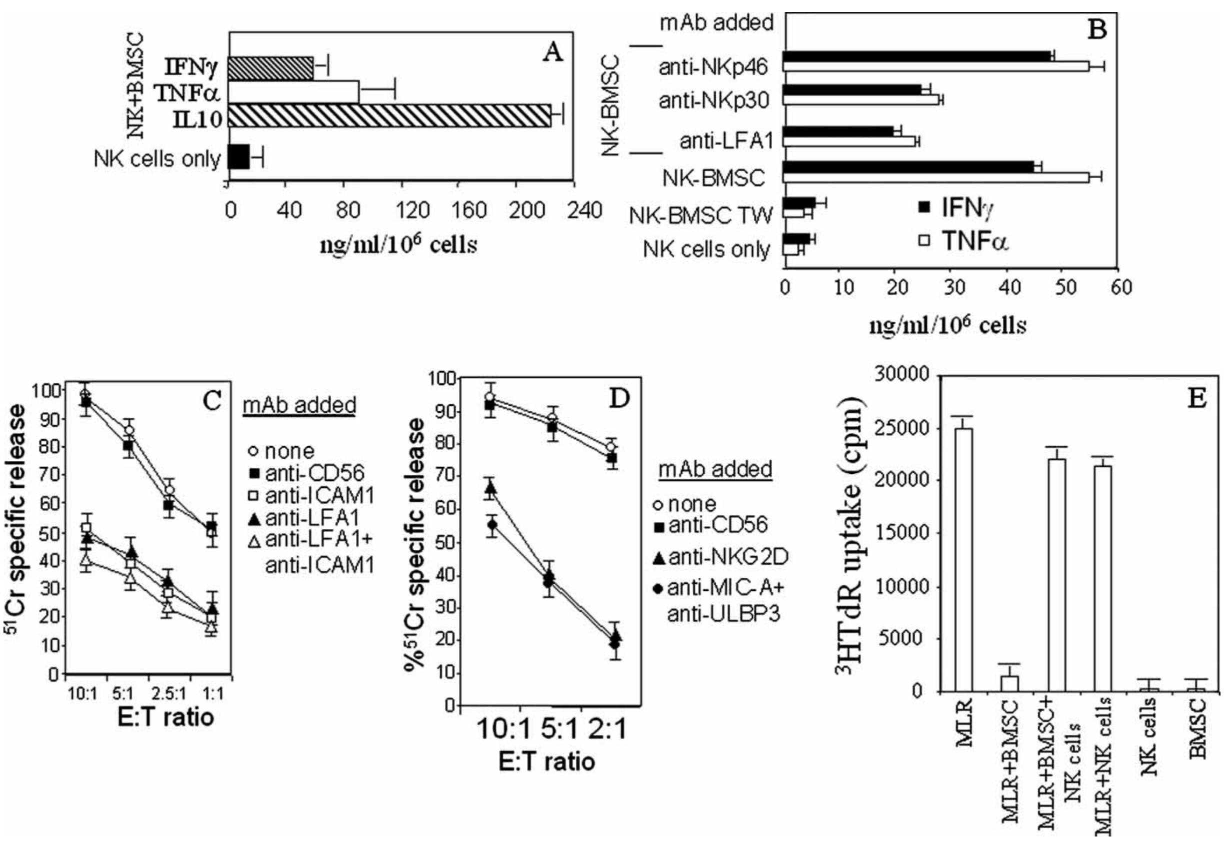

Figure 5. NK cell-mediated cytolytic activity and release of cytokines is triggered by BMSC. Panel (A). Co-culture of *ex vivo* isolated NK cells with BMSC induces production of IFN-γ, TNF-α and IL10. Panel (B). IFN-γ and TNF-α production are dependent on NK–BMSC contact (no production of these cytokines in the absence of NK–BMSC contact: (NK–BMSC TW) and are down-regulated by the covering of LFA1 and/or NKp30 with specific mAb. Results are expressed as ng/ml/$10^6$ cells. Panels (C) and (D). NK cell-mediated lysis of self-BMSC is dependent on LFA1-ICAM1 interaction (C) and the triggering of NKG2D through the engagement of MIC-A or ULBP3 is responsible for the delivering of lethal hit as demonstrated by the use of the specific mAb. Results are expressed as % $^{51}$Cr specific release in a 4 h cytolytic assay at effector (NK cell) target (self APC) (E:T) ratio of 10:1, 5:1, 2:1 (C) and (D) and 1:1 (C). Panel (E). NK cells revert the inhibitory effect of BMSC on lymphocyte proliferation in MLR. Results are expressed as $^3$H-thymidine uptake (cpm) and are the mean ± SD of triplicate samples.

efficient delivery of lethal hit may occur. Killing of BMSC by NK cells was strongly reduced by the addition to the cytolytic assay of the calcium chelator EGTA (not shown), the presence of anti-LFA1 mAb and/or anti-ICAM1 mAb (Figure 5C), or the vacuolar H + -ATPase inhibitor concanamycin A (Poggi et al. 2005) indicating that both calcium increase and release of perforins, conceivably consequent to NK–BMSC binding, are involved (Woo et al. 1998). The addition to the cytolytic assay of any combination of anti-NCR mAb, by contrast of what observed using APC as target cells (see above), did not result in a strong inhibition of BMSC lysis (not shown). Recently, it has been reported that also NKG2D activating receptor (Bauer et al. 1999, Diefenbach et al. 2000, Cosman et al. 2001, Pende et al. 2002, Groh et al. 2002, Poggi et al. 2004) can deliver a triggering signal in cytolytic $CD8^+$T lymphocytes and NK cells, leading to the lysis of tumor target cells that upregulate at the cell surface the NKG2D counter-ligands represented by the MHC-I-related stress inducible molecule A (MIC-A) or (MIC-B) or the UL-16 binding proteins (ULBP1–4). The addition of anti-NKG2D mAb to the cytolytic assay inhibited by 40–80% ($n = 4$) the lysis of BMSC exerted by IL2-activated NK cells and this inhibitory effect was comparable to that obtained with anti-ULBP3 and/or anti-MIC-A mAb were used in combination (Poggi et al. 2005). Indeed, the expression of NKG2D ligands ULBP3, together ULPB1 and 2, and MIC-A on BMSC were confirmed by flow cytometry and microscopic analysis (not shown). The addition of PI-3K inhibitors (LY294002 or wortmannin) reduced the NK cell-mediated lytic effect on BMSC, further confirming the involvement of NKG2D in delivering the lethal hit (Bauer et al. 1999, Poggi et al. 2004). Finally, the addition of anti-HLA class I mAb to the cytolytic assay did not enhance the lytic effect exerted by NK cells on BMSC suggesting that, likewise to what found using APC as target cells, HLA class I do not protect BMSC from aggression of self-NK cells (not shown) (Bauer et al. 1999, Poggi et al. 2004).

*The veto cell effect on mixed lymphocyte reaction mediated by BMSC is down-regulated by NK cells*

Both the spindle type (bona fide MSC) or the polygonal type of stromal cells derived from bone marrow can inhibited MLR when added as a third party (Figure 4C). To determine whether the elimination of these BMSC types by NK cells would result in the downregulation of the BMSC-mediated tolerogenic effect and thus in restoring immune cell response to allogeneic stimulator, we performed MLR in the presence of BMSC either with or without the addition of IL2-activated NK cells. We found that the addition of BMSC to MLR at a responder: BMSC ratio ranging from 1:1 to 10:1 resulted in a strong inhibition of lymphocyte proliferation (75–95% of inhibition; $n = 3$) (Figure 5E). Importantly, this inhibitory effect was almost abolished (90–95% of reconstitution of MLR proliferation) when NK cells were added to MLR/BMSC co-cultures (Figure 5E). NK cells exert a slightly inhibitory effect on MLR (10–15% of inhibition), probably due to consuming of IL2 produced during the interaction between responder and stimulating cells; thus, it is conceivable that the reconstitution of MLR proliferation is essentially due to the elimination of veto effect exerted by BMSC. Polygonal BMSC as well spindle BMSC (containing MSC) were able to inhibit MLR indicating that the veto activity was not confined to MSC; this would suggest that different BMSC can be used *in vivo* to different purposes: BMSC containing MSC to repair tissues and BMSC without differentiating potential to counteract GVHD or in autoimmune diseases. However, both these BMSC types were sensitive to NK cell-mediated lysis implying that NK cells might affect their *in vivo* survival, possibly impairing the BMSC-dependent therapeutic effect.

*Interaction between NK cells and stromal cells derived from skin*

The finding that NK cells can be activated by interaction with self-BMSC prompted us to analyse whether the binding between NK and stromal cells isolated from other tissues can have a similar outcome. Phenotypic analysis of stromal cell populations derived from skin biopsies, thymus and lymph nodes revealed that they do not differ from BMSC for the expression of several surface markers. Indeed, MIC-A and ULBP1, 2 and 3, beside ICAM1 and HLA class I, were present on any kind of stromal cell (not shown). Microscopic analysis showed that these cell populations display, although intracytoplasmic positive for the enzyme 4PH involved in the hydroxylation of collagen, a fibroblast-like morphology similar to that of spindle like BMSC. To determine whether stromal cells isolated from tissues other than bone marrow can exert a veto effect on MLR or on activation of T lymphocytes, we performed MLR or lymphocyte stimulation with PHA or anti-CD3 mAb, in the presence of skin stromal cells (Figure 6A). Unexpectedly, proliferation in MLR was strongly inhibited by the presence of stromal cells (Figure 6A) likewise the lymphocyte activation and proliferation through the CD3/TCR engagement or upon stimulation with PHA (Figure 6B). IL2-activated NK cells killed self-stromal cells upon ligation of NKG2D and LFA1 as reported above for BMSC (Figure 6C). Indeed, the addition of anti-NKG2D or anti-LFA1 mAbs to the cytolytic assay led to a consistent inhibition of NK cell-mediated stromal cell lysis. On the other hand,

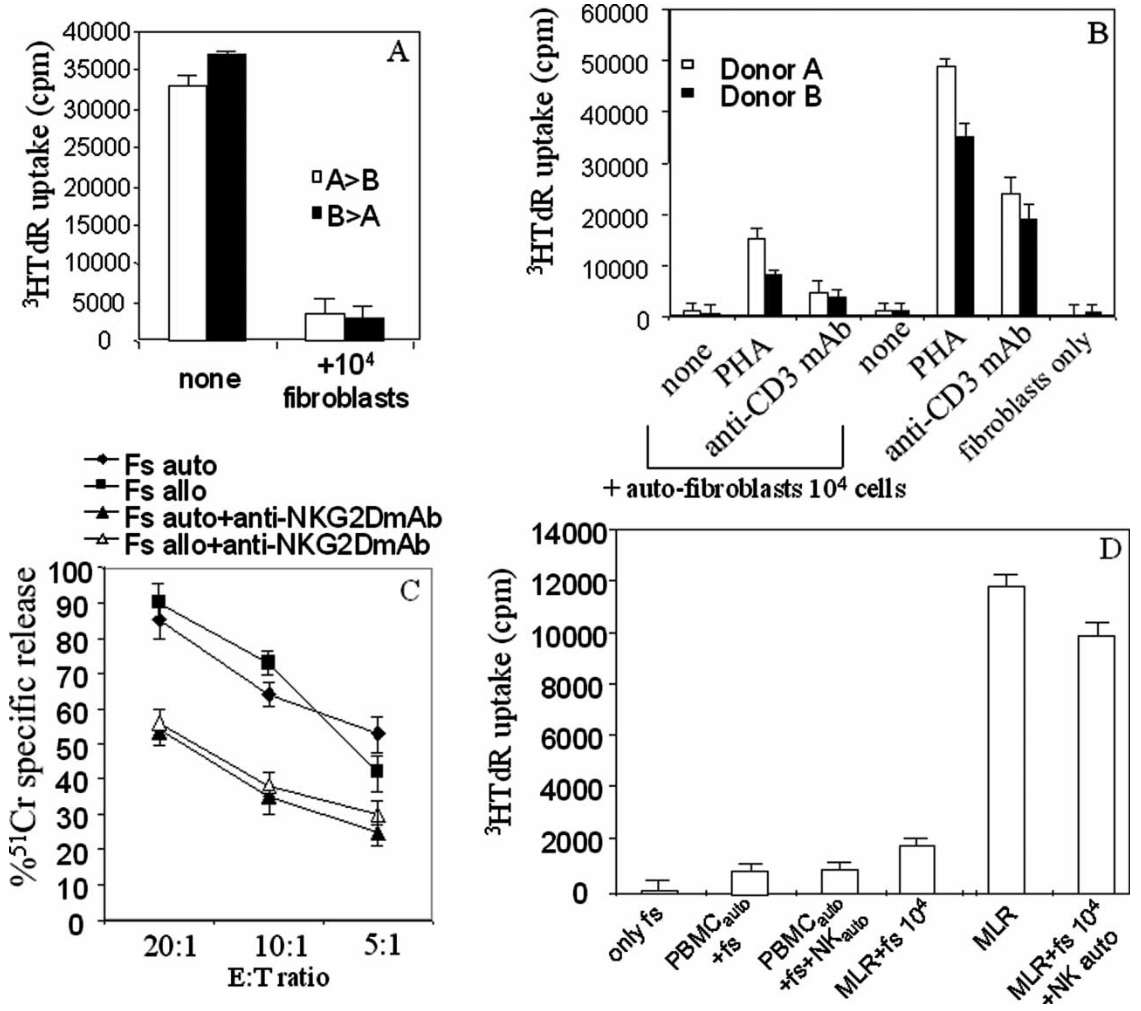

Figure 6. Effect of skin-derived stromal cells on T lymphocyte proliferation: NK cells kill stromal cells and revert their tolerogenic effect. Panel (A). Self-fibroblasts, autologous to responder cells, inhibit T cell proliferation in MLR. Proliferation was analyzed at 7d of culture. Panel (B). A similar inhibitory effect is observed on PHA and CD3-driven T cell proliferation (analyzed on d3). Panel (C). NK cells kill skin stromal cells due to the engagement of NKG2D as the masking of this receptor with specific mAb leads to inhibition of lysis. Panel (D). The addition of NK cells to MLR-skin stromal cells co-culture revert the inhibitory effect mediated by stromal cells. Results are expressed as $^{3}$H-thymidine uptake (cpm) (A, B, D) or as % $^{51}$Cr specific release in a 4 h cytolytic assay at effector (E, NK cell) target (T, self APC) ratio of 20:1, 10:1, 5:1 (C).

anti-NKp30 and/or anti-NKp46 mAbs have a marginal effect on this lysis. Similar to what observed with BMSC, the HLA class I appears to marginally protect stromal cells obtained from skin (not shown). More importantly, NK cells reverted the inhibitory effect on MLR induced by stromal cells (Figure 6D) indicating that NK cells upset the veto effect of stromal cells.

### *Concluding remarks*

NK cell activation against self should be inhibited by the engagement of inhibitory members of the IRS. The present findings, together with other reports (Carbone et al. 1999, Wilson et al. 1999, Parajuli et al. 1999, Spaggiari et al. 2001, Ferlazzo et al. 2002, Pende et al. 2005, Poggi et al. 2005), point out the possibility that NK cells can efficiently interact with self-cells leading to cytokine production and activation of lytic machinery. This, in turn, induces killing and damage of self-APC and/or stromal cells (Figure 7). These findings give rise to two obvious questions: “When does this happen?” and “Which are the regulating mechanism(s) involved?” We can simply hypothesize that NK cells and APC or stromal cells do not encounter themselves in healthy subjects, thus self-aggression will not take place. Against this, it is the finding that NK cells have been found in primary and secondary lymphoid organs (Poggi et al. 1990, Mingari et al. 1991, Ferlazzo et al. 2004) where the interaction between APC and immature/immuno-incompetent (within the thymus) or mature/immuno-competent (in lymph nodes) T cells is essential to avoid self-recognition and mount an efficient immune response. It is conceivable that virus-induced NK cell activation at the site of infection can result in

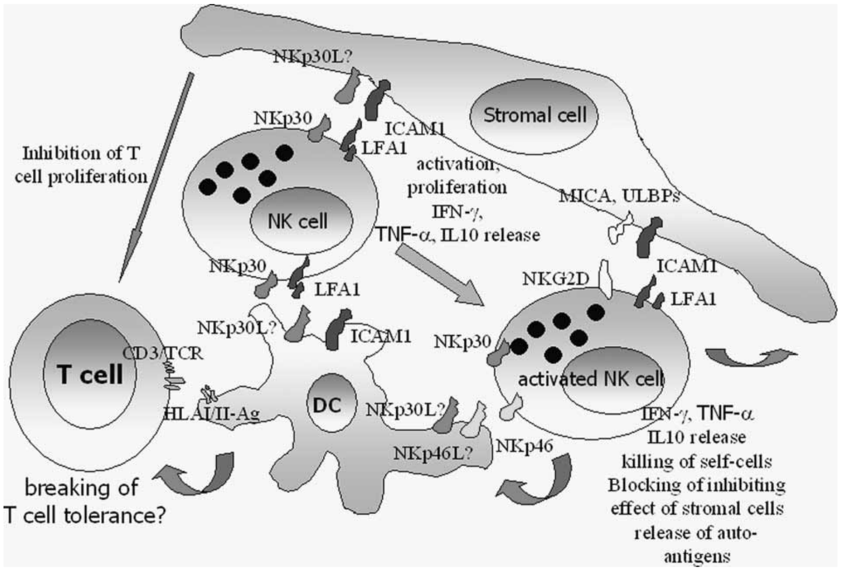

Figure 7. Scheme of interaction between NK cells and self-APC and/or stromal cells. NK cells can interact with specialized APC (DC: dendritic cells) and stromal cells through the engagement of NKp30 and LFA1; this leads to production of pro-inflammatory cytokines as IFN-γ and TNF-α. On the other hand, activated NK cells can damage APC by activation of lytic machinery via NKp30 and NKp46 while NK cells kill stromal cells mainly through NKG2D/MIC-A or NKG2D/ULBPs triggering signal. Both APC and stromal cells have a key role in inducing an optimal or in blocking immune response, respectively. Indeed, APC present correctly the antigen while stromal cells exert a veto-effect on T lymphocyte proliferation. These interactions can be affected by NK cells. Indeed, they can eliminate APC limiting T cell response while they can revert the inhibition of stromal cells on T cell proliferation leading to uncontrolled immune cell response.

the elimination of APC or stromal cells infected by viruses limiting virus life cycle and reducing the spreading of infection. If this is the case, the regulating mechanism(s), which limit the killing activity and pro-inflammatory cytokine production by NK cells, once triggered by virus-infected cells, should be timely switched on. We have shown that the anti-inflammatory cytokine TGFβ is constitutively produced by stromal cells while high amounts of IL10 are detectable in supernatants from NK-stromal cell co-cultures. These cytokines, together with the generation of regulatory T cells may have a role in limiting NK cell-mediated self-reactions. However, we should keep in mind that cytokines are pleiotropic as they influence many elements of immune responses. Indeed, IL10 can also stimulate B cell proliferation despite being one of the most potent inhibitors of cell-mediated immune responses. Similarly, IL2, IL6, TNF-α and IFNs have a well-characterized pro-inflammatory effect, but they can have suppressive activities as well. The discovery of IL23 which stimulates IL17 production, playing a role in autoimmunity or protective response to infections, together with the observation that IL27 can have both pro- and anti-inflammatory effects, further support the notion that the cytokine network have a major role in influencing the outcome of immune response (Hunter 2005). In this context, NK cells bear the correct array of activating surface receptors able to trigger and consequently regulate adaptive immune response against self. Thus, the further study of the functional capabilities of this subpopulation of the innate arm of immune response can lead to a better understanding of the generation of self-aggression and autoimmune disease.

### References

Aggarwal S, Pittenger MF. 2005. Human mesenchymal stem cells modulate allogeneic immune cell responses. Blood 105:1815–1822.

Bauer S, Groh V, Wu J, Steinle A, Phillips JH, Lanier LL, Spies T. 1999. Activation of NK cells and T cells by NKG2D, a receptor for stress-inducible MICA. Science 282:727–729.

Beyth S, Borovsky Z, Mevorach D, Liebergall M, Gazit Z, Aslan H, Galun E, Rachmilewitz J. 2005. Human mesenchymal stem cells alter antigen-presenting cell maturation and induce T cell unresponsiveness. Blood 105:2214–2219.

Carbone E, Terrazzano G, Ruggiero G, Zanzi D, Ottaiano A, Manzo C, Karre K, Zappacosta S. 1999. Recognition of autologous dendritic cells by human NK cells. Eur J Immunol 29:4022–4029.

Cosman D, Mullberg J, Sutherland CL, Chin W, Armitage R, Fanslow W, Kubin M, Chalupny NJ. 2001. ULBPs, novel MHC class-I-related molecules, bind to CMV glicoprotein UL16 and stimulate NK cytotoxicity through the NKG2D receptor. Immunity 14:123–133.

Diefenbach A, Jamieson AM, Liu SD, Shastri N, Raulet DH. 2000. Ligands for the murine NKG2D receptor: Expression by tumor cells and activation of NK cells and macrophages. Nat Immunol 1:119.

Di Nicola M, Carlo-Stella C, Magni M, Milanesi M, Longoni PD, Matteucci P, Grisanti S, Gianni AM. 2002. Human bone marrow stromal cells suppress T-lymphocyte proliferation induced by cellular or nonspecific mitogenic stimuli. Blood 99:3838–3843.

Djouad F, Plence P, Bony C, Tropel P, Apparailly F, Sany J, Noel D, Jorgensen C. 2003. Immunosuppressive effect of mesenchymal stem cells favors tumor growth in allogeneic animals. Blood 102:3837–3844.

Farag SS, Fehniger TA, Ruggeri L, Velardi A, Caligiuri MA. 2002. Natural killer cell receptors: New biology and insights into the graft-versus-leukemia effect. Blood 100:1935–1947.

Ferlazzo G, Thomas D, Lin SL, Goodman K, Morandi B, Muller WA, Moretta A, Munz C. 2004. The abundant NK cells in human secondary lymphoid tissues require activation to express killer cell Ig-like receptors and become cytolytic. J Immunol 172:1455–1462.

Ferlazzo G, Tsang ML, Moretta L, Melioli G, Steinman RM, Munz C. 2002. Human dendritic cells activate resting natural killer (NK) cells and are recognized via the NKp30 receptor by activated NK cells. J Exp Med 195:343–351.

Goodnow CC. 1996. Balancing immunity and tolerance: Deleting and tuning lymphocyte repertoires. Proc Natl Acad Sci 93:2264–2271.

Groh V, Wu J, Yee C, Spies T. 2002. Tumor-derived soluble MIC ligands impair expression of NKG2D and T cell activation. Nature 419:734–738.

von Herrath MG, Harrison LC. 2003. Antigen-induced regulatory T cells in autoimmunity. Nat Rev Immunol 3:223–232.

Hunter CA. 2005. New IL-12-family members: IL23 and Il-27, cytokines with divergent functions. Nat Rev Immunol 5:521–531.

Janaway Jr., CA. 1992. The immune system evolved to discriminate infectious nonself from non-infectious self. Immunol Today 13:11–16.

Lanier LL. 1998. NK cell receptors. Annu Rev Immunol 16:359–393.

Lanzavecchia A, Sallusto F. 2001. Antigen decoding by T lymphocytes: From synapses to fate determination. Nat Immunol 2:487–492.

Le Blanc K. 2003. Immunomodulatory effects of fetal and adult mesenchymal stem cells. Cytotherapy 5:485–489.

Le Blanc K, Rasmusson I, Sundberg B, Gotherstrom C, Hassan M, Uzunel M, Ringden O. 2004. Treatment of severe acute graft-versus host disease with third party haploidentical mesenchymal stem cells. Lancet 363:1439–1441.

Leibson PJ. 1997. Signal transduction during natural killer cell activation: Inside the mind of a killer. Immunity 6:655–661.

Ljunggren HG, Karre K. 1990. In search of the "missing self": MHC molecules and NK cell recognition. Immunol Today 11:237–244.

Long EO. 1999. Regulation of immune responses through inhibitory receptors. Annu Rev Immunol 17:875–904.

Meisel R, Zibert A, Laryea M, Gobel U, Daubener W, Dilloo D. 2004. Human bone marrow stromal cells inhibit allogeneic T-cell responses by indoleamine 2,3-dioxygenase-mediated tryptophan degradation. Blood 103:4619–4621.

Mingari MC, Poggi A, Biassoni R, Bellomo R, Ciccone E, Pella N, Morelli L, Verdiani S, Moretta A, Moretta L. 1991. *In vitro* proliferation and cloning of CD3 − CD16+ cells from human thymocyte precursors. J Exp Med 174:21–26.

Moretta A, Bottino C, Vitale M, Pende D, Biassoni R, Zingari MC, Moretta L. 1996. Receptors for HLA-class I molecules in human natural killer cells. Annu Rev Immunol 14: 619–648.

Moretta A, Bottino C, Vitale M, Pende D, Cantoni C, Zingari MC, Biassoni R, Moretta L. 2001. Activating receptors and co-receptors involved in human natural killer cell-mediated cytolysis. Annu Rev Immunol 19:197–223.

Parajuli P, Nishioka Y, Nishimura N, Singh SM, Hanibuchi M, Nokihara H, Yanagawa H, Sone S. 1999. Cytolysis of human dendritic cells by autologous lymphokine-activated killer cells: Participation of both T cells and NK cells in the killing. J Leukoc Biol 65:764–770.

Pende D, Castriconi R, Romagnani P, Spaggiari GM, Marcenaro S, Dondero A, Lazzeri E, Lasagni L, Martini S, Rivera P, Capobianco A, Moretta L, Moretta A, Bottino C. 2005. Expression of the DNAM-1 ligands nectin-2 (CD112) and poliovirus receptor (CD155) on dendritic cells: Relevance for natural killer/dendritic cells interaction. Blood, in press.

Pende D, Rivera P, Marcenaro S, Chang CC, Biassoni R, Conte R, Kubin M, Cosman D, Ferrone S, Moretta L, Moretta A. 2002. Major histocompatibility complex class I-related chain A and UL16-binding protein expression on tumor cell lines of different histotypes: Analysis of tumor susceptibility to NKG2D-dependent natural killer cell cytoxicity. Cancer Res 62: 6178–6186.

Pittenger MF, Mackay AM, Beck SC, Jaiswal RK, Douglas R, Mosca JD, Moorman MA, Simonetti DW, Craig S, Marshak DR. 1999. Multilineage potential of adult human mesenchymal stem cells. Science 284:143–147.

Podesta M, Benvenuto F, Pitto A, Figari O, Bacigalupo A, Buzzone S, Guida L, Franco L, Paleari L, Bodrato N, Usai C, De Flora A, Zocchi E. 2005. Concentrative uptake of cyclic ADP-ribose generated by BST-1$^+$ stroma stimulates proliferation of human hematopoietic progenitors. J Biol Chem 280:5343–5349.

Poggi A, Biassoni R, Pella N, Paolieri F, Bellomo R, Bertolini A, Moretta L, Mingari MC. 1990. *In vitro* expansion of CD3/TCR-human thymocyte populations that selectively lack CD3 delta gene expression: A phenotypic and functional analysis. J Exp Med 172:1409–1418.

Poggi A, Carosio R, Spaggairi GM, Fortis C, Tambussi G, Dell'Antonio G, Dal Cin E, Rubartelli A, Zocchi MR. 2002. NK cell activation by dendritic cells is dependent on LFA1-mediated induction of calcium–calmodulin kinase II: Inhibition by HIV-1 tat C-terminal domain. J Immunol 168:95–101.

Poggi A, Massaro AM, Negrini S, Pierri I, Balocco M, Michelis G, Aquino S, Albarello A, Gobbi M, Zocchi MR. 2004. Evidence for killing of mesenchymal stem cells (MSC) by autologous natural killer lymphocytes. Blood ASH Annual Meeting Abstracts, November 104:1290.

Poggi A, Prevosto C, massaro AM, Negrini S, Urbani S, Pierri I, Saccardi R, Gobbi M, Zocchi MR. 2005. Interaction between human NK cells and bone marrow stromal cells induces NK cell triggering: Role of NKp30 and NKG2D receptors. J Immunol 175:6352–6360.

Poggi A, Venturino C, Castellani S, Clavio M, Miglino M, Gobbi M, Steinle A, Ghia P, Stella S, Caligaris-Cappio F, Zocchi MR. 2004. Vδ1 T lymphocytes from B-CLL patients recognize ULBP3 expressed on leukemic B cells and upregulated by transretinoic acid. Cancer Res 64: 9172–9179.

Potian JA, Aviv H, Ponzio NM, Harrison JS, Rameshwar P. 2003. Veto-like activity of mesenchymal stem cells: Functional discrimination between cellular responses to alloantigens and recall antigens. J Immunol 171:3426–3434.

Rodriguez Mdel C, Bernad A, Aracil M. 2004. Interleukin-6 deficiency affects bone marrow stromal precursors, resulting in defective hematopoietic support. Blood 103:3349–3354.

Sekiya I, Larson BL, Smith JR, Pochampally R, Cui JG, Prockop DJ. 2002. Expansion of human adult stem cells from bone marrow stroma: Conditions that maximize the yields of early progenitors and evaluate their quality. Stem Cells 20: 530–541.

Spaggiari GM, Carosio R, Pende D, Marcenaro S, Rivera P, Zocchi MR, Moretta L, Poggi A. 2001. NK cell-mediated lysis of autologous antigen-presenting cells is triggered by the engagement of the phosphatidylinositol 3-kinase upon ligation of the natural cytotoxicity receptors NKp30 and NKp46. Eur J Immunol 31:1656–1665.

Trinchieri G. 1989. Biology of natural killer cells. Adv Immunol 47:187–376.

Tse WT, Pendleton JD, Beyer WM, Egalka MC, Guinan EC. 2003. Suppression of allogeneic T-cell proliferation by human marrow stromal cells: Implications in transplantation. Transplantation 75:389–397.

Vodyanik MA, Bork JA, Thomson JA, Slukvin II. 2005. Humman embryonic stem cell-derived $CD34^+$ cells: Efficient production in the co-culture with OP9 stromal cells and analysis of lymphohematopoietic potential. Blood 105:617–626.

Wilson JL, Heffler LC, Charo J, Scheynius A, Bejarano M-T, Ljunggren HG. 1999. Targeting of human dendritic cells by autologous NK cells. J Immunol 163:6365–6370.

Woo JT, Shinohara C, Sakai K, Hasumi K, Endo A. 1998. Isolation, characterization and biological activities of concanamycins as inhibitors of lysosomal acidification. J Antibiot 45:1108–1116.

Woodbury D, Schwarz EJ, Prockop DJ, Black IB. 2000. Adult rat and human bone marrow stromal cells differentiate into neurons. J Neurosci Res 61:364–370.

Zocchi MR, Rubartelli A, Morgavi P, Poggi A. 1998. HIV-1 tat inhibits human natural killer cell function by blocking L-type calcium channels. J Immunol 161:2938–2943.

